# Professional Notes

**Published:** 1887-10-15

**Authors:** 


### Professional Notes.

By a Physician.

Whoever has once or twice plugged the anterior and posterior nares will be glad to know of a remedy which may prevent the necesity for any repetition of the operation. Antypyrin is affirmed not only to be one of several remedies, but to be both prompt and certain in its effects. A watery solution of the strength of 1 to 30 is recommended. A tampon soaked in the fluid is to be brought into contact with as large a portion of the mucous membrane as possible. On the principles recently laid down by Sir Andrew Clark for the rapid cure of hay fever, antypyrin thus applied might be of service in that affection.

---

An easily-applied remedy for corns will be considered generally acceptable. The following is much used by chemists, under a variety of names:—Mix together 1 part of the extract of cannabis sativa, 6 parts of salicylic acid, and 20 parts of collodion. It should be applied with a camel's-hair brush. In four or five days the corn is ready to fall off. A warm foot-bath may then be used to complete the cure.

---

The symptoms manifested by patients are often the sole concern of the medical attendant, although in many instances they are of no more relative importance than the signboard to the contents of a shop. This is especially the case in certain affections whose origin is to be sought in the central nervous system, the brain, or the spinal cord. "Irritative lesions of the nervous centres," says M. Charcot, "like those of nerves, have the power of producing remote trophic disorders in different parts of the body. We may say in a general manner that the skin, the muscles, the joints, the bones, and the viscera may become the seats of various trophic disorders, consequent on lesions of the spinal cord and of the brain." These are facts well known, and rarely forgotten in practice in the case of specific disease, but are very apt to be overlooked in many other instances. Mr. Jonathan Hutchinson recently pointed out the probability that many obscure visceral affections, having well-marked symptoms, are due to herpetic eruptions at the seat of discomfort: herpes of course being merely an indication of some temporary derangement of nervous origin. As an illustration of this general principle of remote trophic disorder in other organs resulting from central nervous lesion, Charcot mentions the case of a "woman 70 years of age, who had been stricken with left hemiplegia, consequent on the formation of a blood-clot in the right cerebral hemisphere. The members of the paralysed side, which at an early period had been contractured, commenced to diminish in bulk, not quite two months after the attack. The muscular wasting affected all parts of the paralysed members in an uniform manner. It was accompanied by a very marked decrease of electrical contractility, and made rapid progress. At the time when the atrophy became evident, the skin of the affected members, on all points subjected to the slightest pressure, presented bullæ which soon gave place to eschars." At the autopsy, hardened sections showed "that the descending fasiculated sclerosis of the left lateral column had been propagated to the anterior cornu of the grey matter of the corresponding side, and had there caused atrophy of a certain number of motor cells." The important observations made during the late American War, as well as those of Ferrier and other recent explorers in the field of cerebro-spinal activity and influence, have thrown a flood of light on affections which were among the profoundest of mysteries at the beginning of the present century. The clear understanding of these variously related facts will save the practitioner from many errors of exposition and practice.

"Little more than a hundred years ago," says Dr. Matthews Duncan, "there were for students in London no human dissections, no specimens, no experiments. The physician had some University education, he defended a thesis, and he walked the hospital. His teachers took a view of their duty diametrically opposite to that taken by modern teachers. Not observation and experiment and induction, but theory and deduction. Above all things they inculcated a system, which was really a flimsy hypothesis, believing it to be as essential to the practitioner's guidance as the pole-star is to the mariner. The theories of Hippocrates, or of Cullen, or some other system, were regarded as embodiments of medical wisdom by those best able to judge. And, though it is now recognised that all medical systems are premature and erroneous generalisations, yet such are not utterly banished from medicine; for we have flourishing still the puny and sickly system of Hahnemann, recognised as an embodiment of medical folly by those best able to judge. Of what is now understood as medical education the surgeon—then in social position much inferior to the physician—had very little. Besides walking the hospitals, he may have assisted at the dissection of a dog."

---

"Many professional men," to quote Dr. Matthews Duncan again, "and those often in remote districts, do, in their humility, fail to recognise their own position as men of science. They seem to think this designation suitable only to men in the great centres of scientific activity, or to such as are Fellows of a Royal Society. But this is a great mistake. The country districts contain many unknown Jenners; and it may afford amusement to attempt to count whether the towns contain proportionally more than the rural districts."

---

"Though medical science," continues Matthews Duncan, "is in some respects remote from medical practice, yet men cannot be good practitioners without being learned in science, the more science applicable the better the practice. The more science in the practitioner, whether that science is applicable or not, the better, *cæteris paribus*, is the practitioner. The mere practical man, often the subject of much silly praise, is either a mistake or he is a humbug."

---

The front-brain, or cerebrum, consists, as everybody knows, of two similar hemispheres, and it is a question often discussed whether or not such an arrangement admits of a *dual consciousness*. So long ago as 1840, Sir Henry Holland raised the question—"Whether some of the aberrations of mind, which come under the name of insanity, are not due to incongruous action of this double structure (the two hemispheres), to which perfect unity of action belongs in the healthy state? The subject is very obscure, and all proof difficult of attainment; but"—he continues—"I think it more probable than otherwise that such inequality may be a cause of some among the many forms of mental derangement. . . . In some cases of hysteria there appear, as it were, two minds; one tending to correct by more just perceptions, feelings, and volitions the aberrations of the other; and the relative power of the two influences varying at different times. . . . I have recently seen a case of which the most marked feature was a frequent and sudden outbreak of passion upon subjects, partly real, partly delusive, but generally without obvious or sufficient reason at the moment; these excesses attended with loud screaming, execrations, and acts of violence in striking or breaking things within reach. Here the patient himself described to me the kind of separate consciousness he had when these violent moods were upon him; his desire, but feelings of inability to resist them; his satisfaction when he felt them to be passing away. It was a painfully exaggerated picture of the struggle between good and ill." Since the date when Sir Henry Holland expressed these questionings, it cannot be said that much new light has been thrown upon this subject of dual consciousness.

